# Assessment of empathy by simulated patients: Adaptation and validation of a new instrument

**DOI:** 10.3205/zma001844

**Published:** 2026-04-15

**Authors:** Pedro Brotons de los Reyes, Montserrat Virumbrales Cancio, Pere Castellvi, Xavier Martínez-Regada, Albert Balaguer

**Affiliations:** 1 Universitat Internacional de Catalunya, School of Medicine and Health Sciences, Department of Medicine, Sant Cugat del Vallès, Spain; 2 Institut de Recerca Sant Joan de Déu, Esplugues de Llobregat, Spain; 3 Instituto de Salud Carlos III, Consorcio de Investigación Biomédica en Red de Epidemiología y Salud (CIBERESP), Madrid, Spain; 4 EAP Amadeu Torner, L'Hospitalet de Llobregat, Barcelona, Spain; 5 Hospital Universitari General de Catalunya, Department of Pediatrics, Barcelona, Spain

**Keywords:** empathy, medical education, medical student, patient simulation, validation study, psychometrics

## Abstract

**Background:**

Medical doctors’ empathy is vital in their interaction with patients, impacting on patient trust and health outcomes. The Consultation and Relational Empathy (CARE) scale helps assess healthcare professionals’ empathy but there is a reasonable doubt about its validity when directly implemented in simulation contexts. This study adapts and validates a version of the CARE scale for simulated patients (Sp-SIMCARE), filling a gap in empathy assessment within medical training simulations.

**Methods:**

The contextual adaptation of the CARE scale for simulated patients followed a four-phase process: 1) preparation of a preliminary adaptation proposal; 2) drafting the first version; 3) piloting the initial version with simulated patients; and 4) refining the final Sp-SIMCARE version. A panel of five experts collaborated with five simulated patients to ensure clarity, relevance, and language equivalence. The validation was conducted using typical primary care scenarios. Simulated patients assessed the performance of undergraduate medical students in four primary patient roles: acute, chronic, with high functional component, and with a hostile attitude. Psychometric parameters that were evaluated included convergent validity (assessed by simulated patients using a global score), acceptability and face validity, homogeneity, and internal reliability.

**Results:**

The adaptation process resulted in a clear, relevant, and comprehensible scale, ensuring uniform understanding among users. Validation involved 95 students in 270 encounters with eight simulated patients. The final version showed convergent validity (Spearman’s rho=0.730, p<0.001), acceptability and face validity (1.96% “Does not apply”/missing values), homogeneity (item-total correlations 0.705-0.865) and reliability (Cronbach’s alpha=0.960).

**Conclusions:**

The Sp-SIMCARE scale appears to be a valid and reliable tool for simulated patients to assess empathy in future doctors during their training from a multidimensional perspective.

## Zusammenfassung

**Hintergrund:**

Das Einfühlungsvermögen von Ärzt:innen ist für deren Interaktion mit Patient:innen von entscheidender Bedeutung und wirkt sich auf das Vertrauen der Patient:innen und die Gesundheitsergebnisse aus. Die CARE-Skala (Consultation and Relational Empathy) hilft bei der Bewertung des Einfühlungsvermögens von medizinischem Fachpersonal, jedoch bestehen begründete Zweifel an ihrer Validität, wenn sie direkt in Simulationskontexten eingesetzt wird. Diese Studie adaptiert und validiert eine Version der CARE-Skala für simulierte Patienten (Sp-SIMCARE) und schließt damit eine Lücke in der Einfühlungsvermögensbewertung innerhalb medizinischer Trainingssimulationen.

**Methoden:**

Die kontextuelle Anpassung der CARE-Skala für simulierte Patient:innen erfolgte in vier Phasen: 1) Vorbereitung eines vorläufigen Anpassungsvorschlags; 2) Entwurf der ersten Version; 3) Erprobung der ersten Version mit simulierten Patient:innen; und 4) Überarbeitung der endgültigen Sp-SIMCARE-Version. Ein Gremium aus fünf Expert:innen arbeitete mit fünf simulierten Patient:innen zusammen, um Klarheit, Relevanz und sprachliche Äquivalenz sicherzustellen. Die Validierung erfolgte anhand typischer Szenarien aus der Primärversorgung. Simulierte Patient:innen bewerteten die Leistung von Medizinstudierernden in vier primären Patient:innenrollen: akut, chronisch, mit hoher funktioneller Komponente und mit einer feindseligen Haltung. Zu den bewerteten psychometrischen Parametern gehörten die konvergente Validität (bewertet durch simulierte Patient:innen anhand einer Gesamtpunktzahl), die Akzeptanz und Augenscheinvalidität, die Homogenität und die interne Reliabilität.

**Ergebnisse:**

Der Anpassungsprozess führte zu einer klaren, relevanten und verständlichen Skala, die ein einheitliches Verständnis unter den Anwendern gewährleistet. An der Validierung nahmen 95 Studierenden in 270 Begegnungen mit acht simulierten Patient:innen teil. Die endgültige Version zeigte konvergente Validität (Spearman’s rho=0,730, p<0,001), Akzeptanz und Augenscheinvalidität (1,96% „trifft nicht zu“/fehlende Werte), Homogenität (Item-Gesamt-Korrelationen 0,705-0,865) und Reliabilität (Cronbach’s Alpha=0,960).

**Schlussfolgerungen:**

Die Sp-SIMCARE-Skala scheint ein valides und zuverlässiges Instrument für simulierte Patient:innen zu sein, um das Einfühlungsvermögen angehender Ärzt:innen während ihrer Ausbildung aus einer multidimensionalen Perspektive zu bewerten.

## 1. Introduction

Medical doctors’ ability to empathize with their patients is essential for establishing trust-based and effective patient-doctor relationships, leading to enhanced clinical outcomes [1]. Despite the emphasis placed on its importance, there is a lack of consensus on the definition of empathy in the specific clinical context. Commonly it is described as a cognitive ability to understand a patient’s inner experiences and perspective, along with the capacity to communicate this understanding [2]. Some authors have distinguished four key components in empathy: emotive (the ability to share the patient’s emotional state), moral (the motivation to act empathetically), cognitive (the capacity to understand the patient’s perspective), and behavioural (the ability to communicate and act in a helpful or therapeutic manner with the patient) [3]. Despite differing models, over recent years most researchers have converged on the importance of clinicians recognizing and expressing awareness of patients’ emotional states though behavioural responses [4], [5], [6]. 

Social neuroscience has recently progressed in identifying that empathy components that can be modified through changes in experiences [7]. Therefore, empathy is a competency that can be taught and learned, especially in the early stages of medical practice. Training in this competence could enhance health care professionals’ and students’ ability to provide effective medical care, while improving patients’ experiences and engagement [8], [9], [10]. Several instruments are available to assess medical doctors' empathy within general practice contexts, such as the Davis’ Interpersonal Reactivity Index (IRI), the Jefferson Scale of Physician Empathy (JSPE), and the Consultation and Relational Empathy (CARE) scale [11], among others. Particularly, the CARE scale evaluates

1. the ability to understand the patient’s situation, perspective and feelings (and their attached meanings);
2. to communicate that understanding and check its accuracy; and
3. to act on that understanding with the patient in a helpful (therapeutic) way [12].

Although the IRI, the JSPE, and other analogous instruments possess pertinent characteristics for the assessment of the emotive and cognitive dimensions of empathy, the CARE scale is distinctive in that it also evaluates the subsequent behavioural response, which is of paramount importance for both patient care and the training of medical students and professionals [13]. 

The CARE scale has undergone translation and validation in primary and specialized care consultations throughout Europe, North America, and Asia [14], [15], [16], [17], [18]. In addition, it has been directly implemented for assessment of empathy in simulation contexts [19], [20], [21], [22], [23]. However, although it seems reasonable that an instrument that has good metric properties in the real context will maintain similar and adequate results in a simulated context, this hypothesis should be tested to ensure its validity. 

The objective of this study was to adapt the Spanish version of the CARE instrument (Sp-CARE) [24] for use among undergraduate medical students in simulated settings and to evaluate its metric properties. 

## 2. Methods

### 2.1. Design and study population

This study took place at the Faculty of Medicine and Health Sciences of the Universitat Internacional de Catalunya (UIC), located in Barcelona, Spain, during the academic course 2022-2023. The participants included fourth-year undergraduate medical students and SPs who had wide experience in the evaluation of “core clinical competencies”, such as interpersonal, communication and listening skills, time management, problem-solving, leadership, and empathy, among others, using standardized empathy measurement tools. The study protocol was approved by the ethics committee at the study centre (registration MED-2022-07). 

### 2.2. The consultation and relational empathy measure

The CARE measure was developed and validated in 2004, showing strong correlation with other empathy-related scales and high internal reliability (Cronbach’s alpha, 0.93) [12]. The scale consists of 10 items scored on a Likert scale from “1” (poor) to “5” (excellent), with higher scores signifying greater levels of empathy within a range from 10 to 50. Developers of the measure suggest that items 1 to 6 are primarily related to the affective aspect of empathy, whereas the remaining items are related to cognitive and behavioural aspects. An option of “does not apply” is also available for each item and can be selected if the item is deemed irrelevant to the consultation. Thus, minimal occurrences of “does not apply” or missing responses demonstrate the patients’ perceived relevance of the item. To ensure practical application, CARE developers recommend permitting a maximum of two “does not apply” or missing responses per measure and disregarding any measure with over two responses of this kind during analysis. For up to two non-applicable or missing responses, they suggest scoring those responses with the average score for the remaining items of the measure, a mean-item score. Validation of the CARE measure in 2004 showed strong convergent high internal reliability (Cronbach’s alpha, 0.93). In 2020, the CARE measure was adapted and validated for use in primary care consultations conducted in the Spanish language (Sp-CARE) [24], demonstrating high acceptability and face validity (1% of non-applicable or missing response), strong homogeneity (corrected item-total correlations >0.30), and robust internal reliability (Cronbach’s alpha, 0.95). 

### 2.3. Contextual adaptation of the Sp-CARE version

The adaptation of the Sp-CARE version to simulation followed a sequential process aimed at maintaining conceptual equivalence within this version and the new Sp-SIMCARE questionnaire. The process consisted of the following phases:

#### 2.3.1. Phase 1: Preparation of a preliminary contextual adaptation proposal

In phase 1, two specialists in development and validation of competence evaluation scales prepared a proposal for preliminary contextual adaptation.

#### 2.3.2. Phase 2: Sp-SIMCARE first version drafting

At this stage, a multi-disciplinary panel of five reviewers who had experience in conducting adaptation and psychometric validation of measurement tools, all of them professors at the faculty of medicine and health sciences at UIC, reviewed all items of the Sp-CARE scale and compared it to each item of the preliminary contextual adaptation proposal. The objective of the comparison was to assess the clarity, relevance, accuracy, and equivalence of meaning of the proposal. 

Each item of the proposal was classified as either

1. conceptually equivalent and easily comprehensible,
2. functionally equivalent but with semantic discrepancies or comprehension difficulties, or
3. of uncertain equivalence.

If an item was classified as (2) or (3), the reviewers were required to clarify the rationale for the mismatch. The first version of Sp-SIMCARE questionnaire was consolidated after resolving the mismatches that had been identified.

#### 2.3.3. Phase 3: Pilot evaluation of Sp-SIMCARE first version with SPs

In phase 3, five SPs assessed the first delivery of the Sp-SIMCARE questionnaire and provided responses to supplementary questions concerning comprehension of all items. These simulated patients also highlighted any irrelevant or offensive wording and were given the opportunity to suggest additional items for inclusion in the questionnaire. Their consultations with simulated patients, coupled with further refinements and improvements that they recommended, prompted the rewording of item 7, which was initially described inappropriately.

#### 2.3.4. Phase 4: Review and refinement of the Sp-SIMCARE final version

During phase 4, the expert panel that had participated in phase 2 debated on the significance of the comments made by the SPs after the pilot evaluation until they reached a consensus, refining the final version of the SP-SIMCARE (see attachment 1 , tables S1 and S2). 

### 2.4. Validation of the Sp-SIMCARE final version

The Sp-SIMCARE questionnaire was validated through encounters of medical students with SPs. Four clinical scenarios, each portraying a common primary care situation, were designed for these encounters. In these scenarios, students interacted with SPs who played the roles of acute, chronic, functional, and patients with a hostile attitude (provoking situations difficult to deal with). To prevent the sharing of information among students, four different clinical cases were developed for each scenario. All clinical cases could be managed within a primary care setting without the necessity for referral to hospital care (see table 1 (Tab. 1) with detailed description of clinical scenarios and characteristics of SPs). Age and gender of undergraduates and patient simulation scenarios were assessed to identify differences in empathy between groups. Psychometric parameters that were evaluated included convergent validity, acceptability and face validity, homogeneity, and internal reliability, as outlined below:

#### 2.4.1. Face validity

The scale’s face validity was evaluated indirectly by the percentage of unanswered non-applicable responses and unanswered items in each survey. Up to two “does not apply” or missing responses were considered acceptable for each survey and substituted by the mean score for the remaining items in compliance with the criteria endorsed by the developers of the CARE measure. 

#### 2.4.2. Convergent validity 

SPs scored undergraduates’ level of empathy globally in response to the direct single question of “Is the student empathetic?” on a Likert scale of 1-10, with greater scores reflecting increased empathy. This global score was considered as the students' final grade in the subject. Therefore, we could consider it as the gold standard for assessing empathy for assessing any student's competency. Convergent validity of scores between the Sp-SIMCARE questionnaire and that global score of empathy was assessed by Pearson or Spearman correlations.

#### 2.4.3. Homogeneity

Homogeneity was examined by corrected item-total correlations, where values above 0.30 predict high correlation [25].

#### 2.4.4. Reliability

Cronbach’s alpha was calculated to assess reliability and determine whether removal of any of the 10 items affected the consistency of the Sp-SIMCARE scale. Alpha values above 0.70 were considered satisfactory [25].

**Figure 1:**
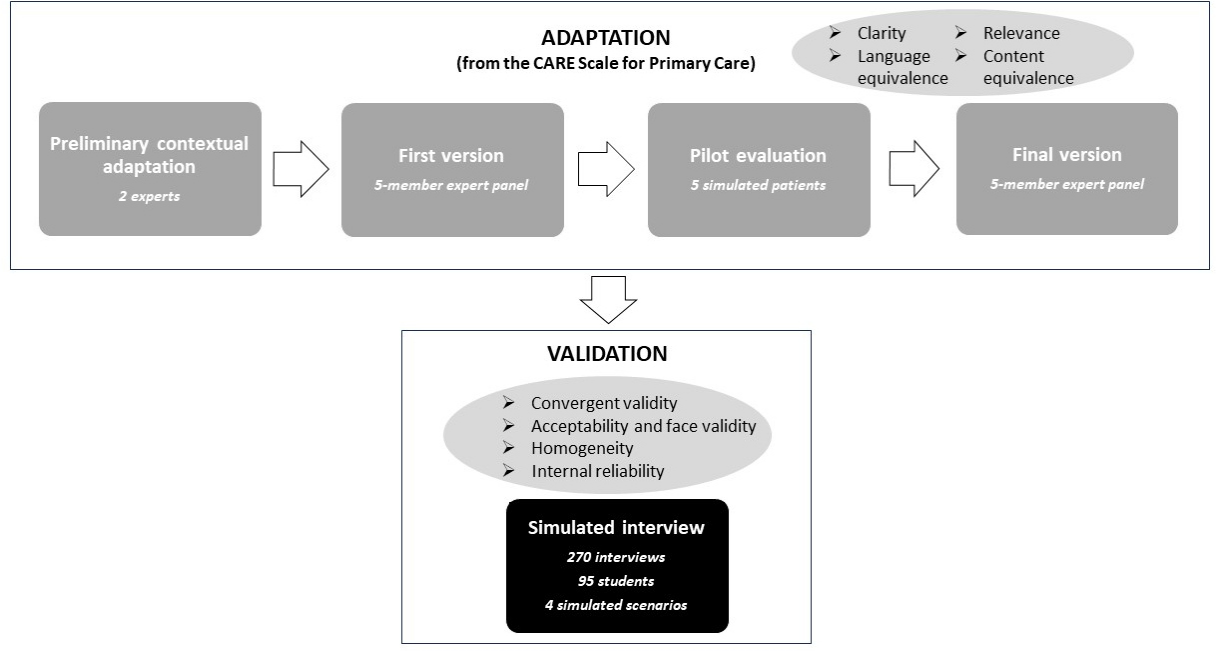
Diagram flow of the Sp-SIMCARE adaptation and validation process

The processes of adaptation and validation of the new instrument are depicted in figure 1 (Fig. 1).

### 2.5. Statistical analysis

Normality of distribution of empathy scores was tested by the Shapiro-Wilk test. The Sp-SIMCARE scores were summarized as mean values and standard deviations or median and interquartile range values according to the normal or skewed distribution of data, respectively, and categorical variables were described as proportions. Comparison of scores by gender, age group, and simulated scenario was performed using the student t or the Mann-Whitney test (two-group comparison) and the ANOVA or the Kruskal-Wallis test (multi-group comparisons). The level of significance was set at 5% (p<0.05). Data analysis was performed using the statistical package Stata v. 15. All the identifying information of the students was duly anonymized.

## 3. Results

The contextual adaptation process ensured the Sp-SIMCARE items were relevant, clearly worded, understandable, and had equivalent meaning for all users. A total of 95 students, 83% of whom were under 26 years old and 63.2% females, and eight SPs encountered in the four scenarios for the validation of the new instrument. Due to various contingencies, primarily related to students’ absences, 297 out of the scheduled 380 encounters between students and SPs were completed (see table 2 (Tab. 2)). Among the 297 SP evaluations of these encounters, 27 (9.1%) had three or more non-applicable or missing responses and were excluded from analysis, in line with the criteria recommended by the CARE developers. 

The validation procedure eventually involved the examination of students’ performance in 270 encounters that resulted in a total of 2,700 items of the Sp-SIMCARE being scored (see table 2 (Tab. 2)). The median score was 32.5 (IQR, 29.0-37.0), with scores ranging from 10 to 50. Most of the responses to the individual items of the scale were scored as either “good” (45.2%) or “very good” (34.1%). There were no statistically significant in levels of empathy amongst the participants, based on age, gender or the type of simulated patient (see table 3 (Tab. 3)).

The Sp-SIMCARE questionnaire demonstrated high face validity: only 53 (1.96%) out of the total 2,700 responses were “does not apply” choices or left blank (see table 2 (Tab. 2)). Out of 270 surveys, there were 21 (7.8%) with two non-applicable or blank responses, and 11 (4.1%) with one of such responses. The encounters revealed that such responses were most frequent among difficult-to-deal-with patients (32/470, 6.81%), whereas the proportion was notably lower for chronic patients (19/750, 2.53%), and absent for acute or functional patients (see attachment 1 , table S2). It was noted that a significant proportion of respondents either selected “does not apply” or left blank responses for items 9 “assist you in taking charge” (26/270, 9.6%) and 10 “collaborate with you to formulate a plan of action” (23/270, 8.5%). The proportion of non-applicable or missing responses to items 7 (4/270, 1.5%) and 8 (3/270, 1.0%) was much lower. Items 1 to 6 were scored in all surveys. 

A significant positive correlation (Spearman’s rho coefficient, 0.730; p<0.001) was found between the scores provided by the simulated patients by the Sp-SIMCARE scale and in response to the query “Is the student empathetic?”, after confirming the skewed distribution of scores. Corrected item-total correlations ranged from 0.797 to 0.869, and Cronbach’s alpha value for the scale was 0.960, with values for individual items falling within the range of 0.954-0.957 (see table 4 (Tab. 4)).

## 4. Discussion

This study presents evidence of high validity and reliability of a new questionnaire, adapted from the well-known CARE instrument, which standardises the assessment of relational empathy in the context of clinical situations with simulated patients. The psychometric results support this tool as suitable, valid, and potentially useful for use with medical students in such settings. 

Based on the results of the study, the new questionnaire demonstrated high acceptability with only 1.96% of “not applicable or blank” responses, which is comparable to the 1% observed in the validation of the previous Sp-CARE version [24]. This outcome indicates that the interactions generated in the simulation scenarios were realistic, varied, and effectively evaluated using the new questionnaire. Previous studies conducted in other primary care settings have documented a wide spectrum of acceptability of the original CARE measure and derived versions, with some works reporting similarly low rates of non-applicant or missing responses [16], [24] and others describing much higher percentages [14], [17]. The scenarios involving simulated patients difficult to deal with received the highest percentage of “not applicable or blank” responses (6.8%). These responses were concentrated in two specific items: 9 (9.6%) and 10 (8.5%). Those percentages were consistent with similar proportions of face validity for items 9 and 10 that were reported in earlier research on the CARE measure [12], [14], [15], [16]. Low face validity for the two items was predictable since management of emotions and patient containment prevailed over the expected performance of students when dealing with conflictive patients. In this regard, a previous study even postulated that item 10 should be excluded from scoring when assessing relational empathy, as it may not be an accurate determinant of a medical doctor's empathy but rather reflect shared decision making [26]. 

The median Sp-SIMCARE score obtained from our study population (32.5) was significantly lower than mean or median scores (above 40) previously published in CARE validation or implementation studies conducted in European primary care settings [12], [16]. The notable variation in scores between our and other European studies could be explained by the different study populations under evaluation: our study assessed the performance of undergraduate medical students who had limited prior experience interacting with simulated patients, while other studies evaluated the performance of primary care medical doctors who commonly have regular and intense interactions with their patients and, as a result, are more likely to exhibit empathetic competency. Interestingly, no significant variations were found in Sp-SIMCARE scores based on the gender of undergraduate medical students. This outcome aligns with prior studies, which suggested that the scores by the CARE measure were not substantially affected by either the gender of medical professionals or consultation characteristics [13], [14], [15], [24]. Furthermore, although the population studied here (fourth-year students) may have had an initial imbalance in empathy in favour of women on entering medical school, this difference could have been compensated for by the training received throughout the years of study, as we showed in [27]. 

The Sp-SIMCARE questionnaire displayed robust convergence (Spearman’s rho coefficient, 0.730) with the scores provided by simulated patients in response to the explicit query “Is the student empathetic?”, which were used for global evaluation of the simulation exercise. This outcome indicates that the novel scale measures students’ empathy levels in a manner that aligns with the comprehensive assessment of empathy conducted by simulated patients in the four most common types of clinical patients (chronic, acute, functional, and difficult-to-deal with), giving validity to the use of the Sp-SIMCARE scale in different simulation contexts. Overall, corrected item-total correlation values (>0.797) and Cronbach’s alpha values (>0.954) were high in our study and revealed strong homogeneity and reliability of the new tool, in line with values reported for these measures in other previous validation studies of CARE versions [13], [14], [15], [24]. 

The present work logically presents some methodological strengths and limitations. Among the strengths, it is worth highlighting the meticulous sequential process followed to adapt Sp-CARE to the simulation context. Furthermore, the fact that it was tested in different pathology scenarios and by simulated patients with different profiles suggests that the tool has a good usability. In this sense, the scenarios, which had undergone a prior design and validation process by a committee of experts, considered not only different clinical situations, but also different types of patients, including a specific scenario with hostile SPs characterized by lack of cooperation or high aggressiveness. One limitation of the study is the absence of an objective gold-standard of relational empathy. Therefore, to assess the convergent validity of the Sp-SIMCARE questionnaire, it was necessary to compare its results with a proxy for the gold standard. In this case, we did not use a validated survey to measure empathy but instead relied on the simulated patient’s self-reported perception of empathy during the clinical encounter. This served as our external standard or criterion. While some may consider this a limitation, it is a commonly used procedure in similar cases. On the other hand, the study population size was small, which restricted the possibility of exploratory and confirmatory factor analyses. Additionally, the origin of the study population, consisting of fourth-year student volunteers, suggests the need for caution when generalizing the results.

The Sp-SIMCARE questionnaire's focus on the relational aspects of empathy provides an advantage. It assesses how empathy translates into concrete actions during simulated interactions, offering valuable insight beyond a subjective and emotional understanding. The questionnaire is particularly sensitive to the complexities of interacting with varied scenarios and patients, including the most difficult ones. Adaptability is crucial in medical training, particularly when testing empathy in challenging clinical situations, such as with conflictive patients. To be highlighted, the use of simulated environments for questionnaire validation is a strategic choice. It provides a controlled and safe environment, ensuring a consistent and fair assessment for all students, especially those who are still learning. This methodology addresses the practical barriers associated with obtaining direct feedback from patients by providing a structured and reproducible assessment. Importantly, the validation of Sp-SIMCARE in advanced medical students suggests its potential usefulness for assessing relational empathy in other health care professional groups, although further studies are needed to confirm this.

## 5. Conclusion

In summary, the Sp-SIMCARE questionnaire proved to be psychometrically valid and reliable for evaluation of undergraduate medical students by simulated patients. The questionnaire’s uniqueness lies in its ability to measure the relational dimension of empathy, providing a practical tool for assessing this competence. The use of this new tool could potentially assist in the design and implementation of interventions aimed at fostering empathy in future doctors throughout their training.

## Acknowledgements

The authors acknowledge the contribution of simulated patients and students enrolled in the Degree in Medicine of the Universitat International de Catalunya that participated in this study.

## Notes

### Ethics approval and consent to participate

The study protocol was approved by the Ethics Committee at the study centre (registration MED-2022-07). The approval of the protocol waived the requirement to obtain informed consent from participants in the study.

### Funding

This study was partially funded by a competitive grant for medical education research projects awarded by the Sociedad Española de Educación Médica (SEDEM).

### Authorship

Pedro Brotons de los Reyes and Montserrat Virumbrales Cancio share the first authorship.

### Author contributions

- PB : Data analysis, interpretation of results, draft manuscript preparation, final manuscript approval
- MV: Study conception and design, data collection, interpretation of results, final manuscript approval
- PC: Interpretation of results, final manuscript approval
- XM: Data collection, interpretation of results, final manuscript approval
- SD: Data collection, interpretation of results, final manuscript approval
- AB: Study conception and design, study management, interpretation of results, final manuscript approval

### Authors’ ORCIDs

- Pedro Brotons de los Reyes: [0000-0002-2399-7320]
- Montserrat Virumbrales Cancio: [0000-0003-3541-2948]
- Pere Castellvi: [0000-0002-3920-8576]
- Xavier Martinez-Regada: [0009-0009-5013-7738]
- Albert Balaguer: [0000-0002-5222-8635]

## Competing interests

The authors declare that they have no competing interests. 

## Supplementary Material

Supplementary material

## Figures and Tables

**Table 1:**
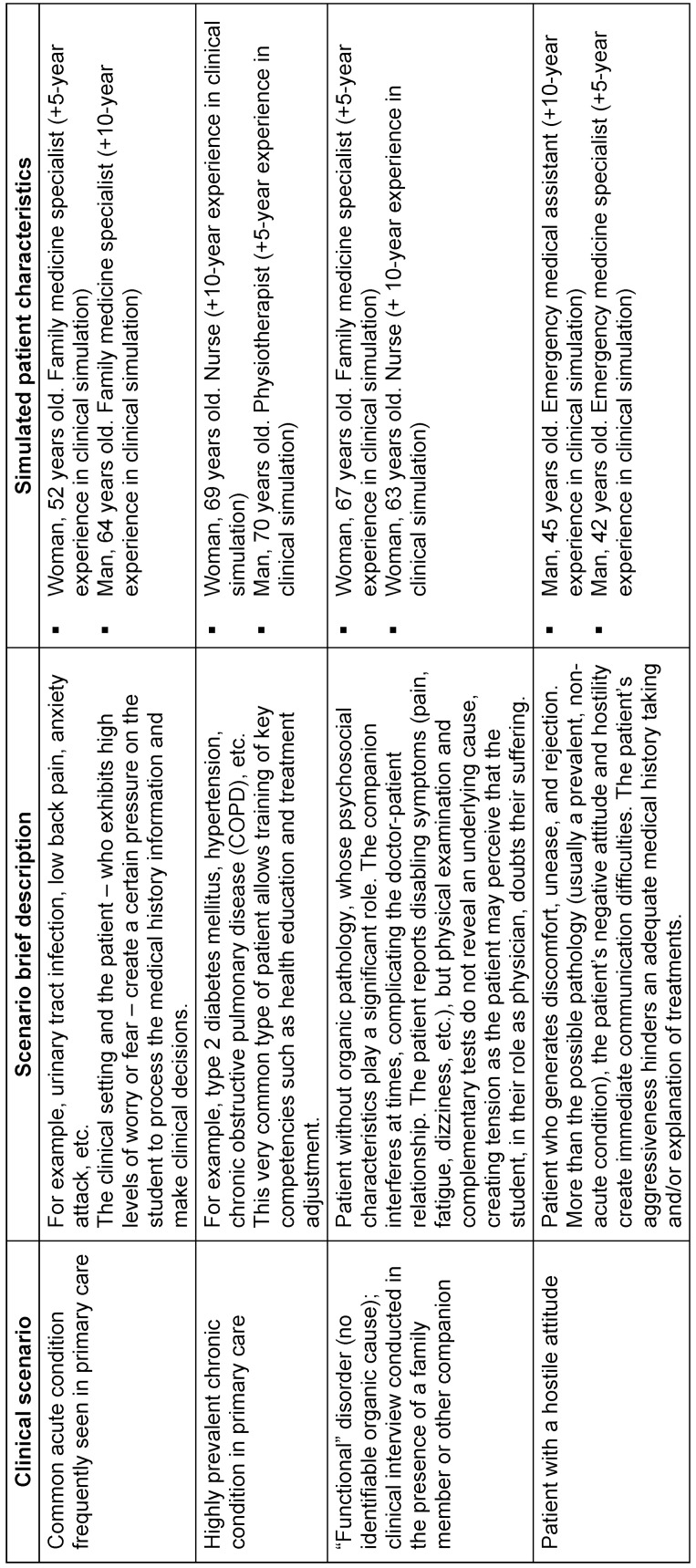
Overview of clinical scenarios and simulated patient characteristics

**Table 2:**
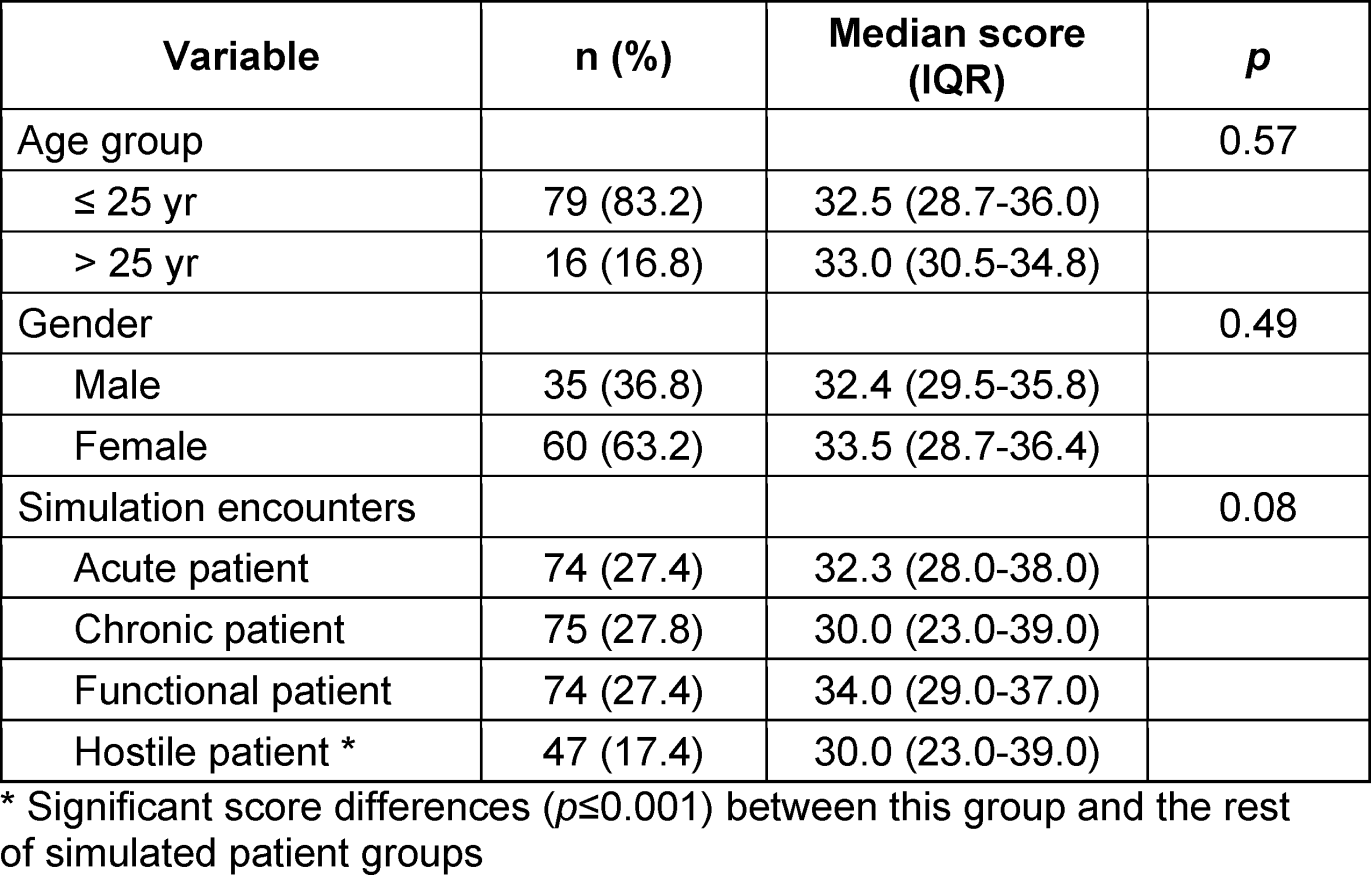
Medical students’ demographics and Sp-SIMCARE scores by age group, gender, and simulated scenario

**Table 3:**
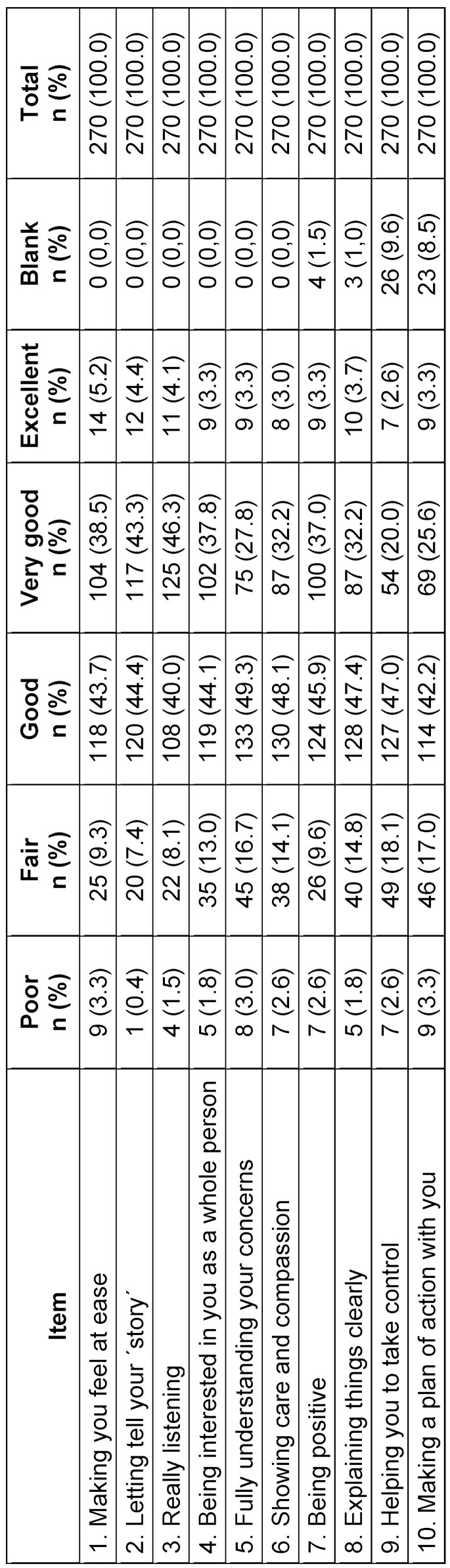
Sp-SIMCARE scores for medical student performance

**Table 4:**
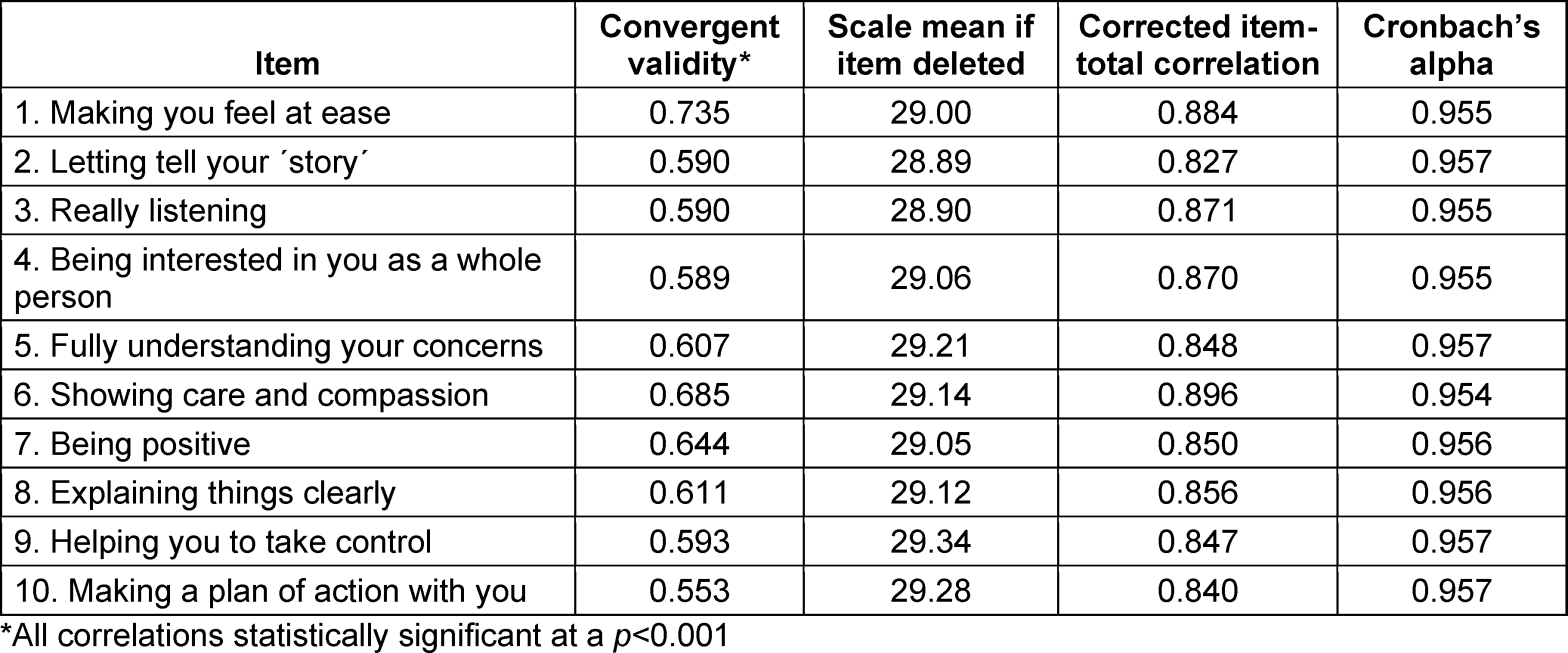
Convergent validity, homogeneity, and internal reliability of the Sp-SIMCARE questionnaire
