# Supplementary material for: Assessment of empathy by simulated patients: Adaptation and validation of a new instrument
Source: GMS J Med Educ. 2026 Apr 15;43(4):Doc50. doi: 10.3205/zma001844 (PMC13124498; doi:10.3205/zma001844)
Supplement: Supplementary material [file JME-43-50-s-001.pdf]

Attachment 1: Supplementary material

Attachment 1 to Brotons de los Reyes P, Virumbrales Cancio M, Castellvi P, Martinez-Regada X, Balaguer A. *Assessment of empathy by simulated patients: adaptation and validation of a new instrument.* GMS J Med Educ. 2026;43(4):Doc50. DOI: 10.3205/zma001844

Table S1: Sp-SIMCARE questionnaire items

No. item	Sp-CARE	Sp-SIMCARE
1	Hacerte sentir cómodo/a (presentándose, indicando su cargo, siendo amable y acogedor/a, tratándose con respeto; no de forma fría o brusca)	*Me hace sentir cómodo/a (se presenta, indica su cargo, es amable y acogedor/a, me trata con respeto y no de forma fría o brusca)*
2	Dejarte contar tu "historia" (dándote tiempo para describir con detalle tu estado de salud con tus propias palabras; sin interrumpirte, meterte prisa o desviarte del tema)	*Me deja contar mi "historia" (me da tiempo para describir con detalle mi estado de salud con mis propias palabras; sin interrumpir, meter prisa o desviarme del tema)*
3	Escucharte atentamente (prestando toda su atención a lo que le decías; sin mirar las notas o el ordenador mientras hablabas)	*Me escucha atentamente (presta toda su atención a lo que digo. No mira las notas o el ordenador mientras hablo)*
4	Interesarse por ti como persona (preguntando/conociendo detalles relevantes sobre tu vida, tu situación; sin tratarte "como un número")	*Se interesa por mí como persona (me pregunta o conoce detalles relevantes sobre mi vida y mi situación. No me trata "como un número")*
5	Comprender completamente tus preocupaciones (comunicando que ha comprendido tus preocupaciones y ansiedades con exactitud; sin pasar por alto ni restar importancia a nada)	*Comprende completamente mis preocupaciones (deja claro que ha entendido mis preocupaciones y ansiedades con exactitud. No pasa por alto ni resta importancia a nada)*
6	Mostrar interés y compasión (mostrándose genuinamente preocupado/a, conectando contigo a nivel humano; sin ser indiferente o distante)	*Muestra interés y compasión (se muestra genuinamente preocupado/a y conecta conmigo a nivel humano. No se muestra indiferente o distante)*
7	Ser positivo/a (teniendo un enfoque positivo y una actitud positiva; siendo honesto/a pero no negativo/a acerca de tu situación)	*Es positivo/a (tiene un enfoque y actitud positivas; es honesto/a pero no negativo/a acerca de mi situación)*
8	Explicar las cosas con claridad (respondiendo totalmente a tus preguntas; explicando con claridad, dando la información adecuada; sin vaguedades)	*Explica las cosas con claridad (responde totalmente a mis preguntas; se explica con claridad; da la información adecuada evitando tecnicismos. Se expresa sin vaguedades)*
9	Ayudarte a tomar el control (analizando contigo qué puedes hacer para mejorar tu salud por ti mismo/a; alentándote en lugar de "sermoneándote")	*Me ayuda a tomar el control de la situación (analiza conmigo qué puedo hacer para mejorar mi salud por mií mismo/a. Me anima en lugar de sermonear)*
10	Diseñar un plan de acción contigo (valorando las opciones, implicándote en las decisiones en la medida que desees implicarte; sin ignorar tus opiniones)	*Diseña un plan de acción conmigo (valora conmigo las opciones y me implica en las decisiones en la medida que yo deseo implicarme. No ignora mis opiniones)*

Table S2: English translation of the Sp-SIMCARE questionnaire items

No. item	Sp-SIMCARE	Sp-SIMCARE (English translation)
1	*Me hace sentir cómodo/a (se presenta, indica su cargo, es amable y acogedor/a, me trata con respeto y no de forma fría o brusca)*	*He/She makes me feel comfortable (introduces him/herself, indicates his/her position, is friendly and welcoming, treats me with respect and not in a cold or abrupt manner)*
2	*Me deja contar mi "historia" (me da tiempo para describir con detalle mi estado de salud con mis propias palabras; sin interrumpir, meter prisa o desviarme del tema)*	*He/She lets me tell my "story" (he gives me time to describe in detail my health condition in my own words; without interrupting, rushing or getting off topic).*
3	*Me escucha atentamente (presta toda su atención a lo que digo. No mira las notas o el ordenador mientras hablo)*	*He/She listens to me attentively (pays full attention to what I say. He does not look at the notes or the computer while I am talking).*
4	*Se interesa por mí como persona (me pregunta o conoce detalles relevantes sobre mi vida y mi situación. No me trata "como un número")*	*He/She is interested in me as a person (asks me or knows relevant details about my life and situation. Does not treat me "as a number")*
5	*Comprende completamente mis preocupaciones (deja claro que ha entendido mis preocupaciones y ansiedades con exactitud. No pasa por alto ni resta importancia a nada)*	*He/She completely understands my concerns (makes it clear that he has understood my concerns and anxieties accurately. Does not overlook or downplay anything).*
6	*Muestra interés y compasión (se muestra genuinamente preocupado/a y conecta conmigo a nivel humano. No se muestra indiferente o distante)*	*He/She shows interest and compassion (shows genuine concern and connects with me on a human level. Does not appear indifferent or distant).*
7	*Es positivo/a (tiene un enfoque y actitud positivas; es honesto/a pero no negativo/a acerca de mi situación)*	*He/She is positive (has a positive outlook and attitude; is honest but not negative about my situation)*
8	*Explica las cosas con claridad (responde totalmente a mis preguntas; se explica con claridad; da la información adecuada evitando tecnicismos. Se expresa sin vaguedades)*	*He/She explains things clearly (answers my questions fully; explains himself/herself clearly; gives the right information avoiding technicalities; expresses himself/herself without vagueness)*
9	*Me ayuda a tomar el control de la situación (analiza conmigo qué puedo hacer para mejorar mi salud por m~~i~~í mismo/a. Me anima en lugar de sermonear)*	*He/She helps me take control of the situation (discusses with me what I can do to improve my health on my own. Encourages me instead of lecturing me).*
10	*Diseña un plan de acción conmigo (valora conmigo las opciones y me implica en las decisiones en la medida que yo deseo implicarme. No ignora mis opiniones)*	*He/she devises a plan of action with me (weighs options with me and involves me in decisions to the extent that I wish to be involved. Does not ignore my opinions)*

Table S3: Sp-SIMCARE scores of medical student performance by simulated patient type (acute, chronic, functional, and hostile)

		Poor	Fair	Good	Very good	Excellent	Blank	Total
**Simulated patient type**	**Item**	**n (%)**	**n (%)**	**n (%)**	**n (%)**	**n (%)**	**n (%)**	**n (%)**
Acute patient	1.	3 (4.0)	9 (12.2)	33 (44.6)	22 (29.7)	7 (9.5)	0 (0.0)	74 (100.0)
	2.	0 (0.0)	5 (6.7)	31 (41.9)	31 (41.9)	7 (9.5)	0 (0.0)	74 (100.0)
	3.	1 (1.4)	7 (9.5)	32 (43.2)	28 (37.8)	6 (8.1)	0 (0.0)	74 (100.0)
	4.	0 (0.0)	11 (14.9)	37 (50.0)	22 (29.7)	4 (5.4)	0 (0.0)	74 (100.0)
	5.	3 (4.0)	12 (16.2)	35 (47.3)	21 (28.4)	3 (4.0)	0 (0.0)	74 (100.0)
	6.	1 (1.4)	12 (16.2)	34 (45.9)	23 (31.1)	4 (5.4)	0 (0.0)	74 (100.0)
	7.	2 (2.7)	10 (13.5)	31 (41.9)	24 (32.4)	6 (8.1)	1 (1.4)	74 (100.0)
	8.	1 (1.4)	15 (20.3)	30 (40.5)	23 (31.1)	5 (6.8)	0 (0.0)	74 (100.0)
	9.	4 (5.4)	21 (28.4)	29 (39.2)	17 (23.0)	2 (2.7)	1 (1.4)	74 (100.0)
	10.	5 (6.8)	16 (21.6)	25 (33.8)	22 (29.7)	6 (8.1)	0 (0.0)	74 (100.0)
Chronic patient	1.	0 (0.0)	2 (2.7)	38 (50.7)	32 (42.7)	3 (4.0)	0 (0.0)	75 (100.0)
	2.	0 (0.0)	6 (8.0)	37 (49.3)	30 (40.0)	2 (2.7)	0 (0.0)	75 (100.0)
	3.	0 (0.0)	4 (5.3)	36 (48.0)	33 (44.0)	2 (2.7)	0 (0.0)	75 (100.0)
	4.	0 (0.0)	5 (6.7)	38 (50.7)	30 (40.0)	2 (2.7)	0 (0.0)	75 (100.0)
	5.	0 (0.0)	8 (10.7)	43 (57.3)	22 (29.3)	2 (2.7)	0 (0.0)	75 (100.0)
	6.	0 (0.0)	7 (9.3)	47 (62.7)	19 (25.3)	2 (2.7)	0 (0.0)	75 (100.0)
	7.	0 (0.0)	0 (0.0)	47 (62.7)	25 (33.3)	3 (4.0)	0 (0.0)	75 (100.0)
	8.	0 (0.0)	7 (9.3)	37 (49.3)	28 (37.3)	3 (4.0)	0 (0.0)	75 (100.0)
	9.	0 (0.0)	6 (8.0)	41 (54.7)	16 (21.3)	2 (2.7)	10 (13.3)	75 (100.0)
	10.	0 (0.0)	7 (9.3)	36 (48.0)	21 (28.0)	2 (2.7)	9 (12.0)	75 (100.0)

		Poor	Fair	Good	Very Good	Excellent	Blank	Total
Functional patient	1.	1 (1.4)	4 (5.4)	29 (39.2)	39 (52.7)	1 (1.4)	0 (0.0)	74 (100.0)
	2.	0 (0.0)	4 (5.4)	32 (43.2)	38 (51.4)	0 (0.0)	0 (0.0)	74 (100.0)
	3.	0 (0.0)	5 (6.7)	23 (31.1)	46 (62.2)	0 (0.0)	0 (0.0)	74 (100.0)
	4.	0 (0.0)	6 (8.1)	36 (48.7)	32 (43.2)	0 (0.0)	0 (0.0)	74 (100.0)
	5.	0 (0.0)	15 (20.3)	39 (52.7)	19 (25.7)	1 (1.4)	0 (0.0)	74 (100.0)
	6.	0 (0.0)	4 (5.4)	40 (54.1)	30 (40.5)	0 (0.0)	0 (0.0)	74 (100.0)
	7.	0 (0.0)	8 (10.8)	29 (39.2)	37 (50.0)	0 (0.0)	0 (0.0)	74 (100.0)
	8.	1 (1.4)	8 (10.8)	45 (60.8)	20 (27.0)	0 (0.0)	0 (0.0)	74 (100.0)
	9.	1 (1.4)	18 (24.3)	46 (62.2)	9 (12.2)	0 (0.0)	0 (0.0)	74 (100.0)
	10.	1 (1.4)	17 (23.0)	41 (55.4)	15 (20.3)	0 (0.0)	0 (0.0)	74 (100.0)
Hostile patient	1.	5 (10.6)	10 (21.3)	18 (38.3)	11 (23.4)	3 (6.4)	0 (0.0)	47 (100.0)
	2.	1 (2.1)	5 (10.6)	20 (42.6)	18 (38.3)	3 (6.4)	0 (0.0)	47 (100.0)
	3.	3 (6.4)	6 (12.8)	17 (36.2)	18 (38.3)	3 (6.4)	0 (0.0)	47 (100.0)
	4.	5 (10.6)	13 (27.7)	8 (17.0)	18 (38.3)	3 (6.4)	0 (0.0)	47 (100.0)
	5.	5 (10.6)	10 (21.3)	16 (34.0)	13 (27.7)	3 (6.4)	0 (0.0)	47 (100.0)
	6.	6 (12.8)	15 (31.9)	9 (19.2)	15 (31.9)	2 (4.3)	0 (0.0)	47 (100.0)
	7.	5 (10.6)	8 (17.0)	17 (36.2)	14 (29.8)	0 (0.0)	3 (6.4)	47 (100.0)
	8.	3 (6.4)	10 (21.3)	16 (36.2)	16 (34.0)	2 (4.3)	0 (0.0)	47 (100.0)
	9.	2 (4.3)	4 (8.5)	11 (23.4)	12 (25.5)	3 (6.4)	15 (31.9)	47 (100.0)
	10.	3 (6.4)	6 (12.8)	12 (25.5)	11 (23.4)	1 (2.1)	14 (29.8)	47 (100.0)
